# Anti-Platelet Aggregation and Vasorelaxing Effects of the Constituents of the Rhizomes of *Zingiber officinale*

**DOI:** 10.3390/molecules17088928

**Published:** 2012-07-26

**Authors:** Yu-Ren Liao, Yann-Lii Leu, Yu-Yi Chan, Ping-Chung Kuo, Tian-Shung Wu

**Affiliations:** 1 Department of Chemistry, National Cheng Kung University, Tainan 701, Taiwan; Email: l3892101@mail.ncku.edu.tw; 2 Graduate Institute of Natural Products, Chang Gung University, Taoyuan 333, Taiwan; Email: ylleu@mail.cgu.edu.tw; 3 Department of Biotechnology, Southern Taiwan University, Tainan 710, Taiwan; Email: yuyichan@mail.stut.edu.tw; 4 Department of Biotechnology, National Formosa University, Yunlin 632, Taiwan; Email: pcckuoo@sunws.nfu.edu.tw; 5 Department of Pharmacy, China Medical University, Taichung 404, Taiwan; 6 Chinese Medicinal Research and Development Center, China Medical University and Hospital, Taichung 404, Taiwan

**Keywords:** anti-platelet aggregation, vasorelaxing, Zingiberaceae, gingerol, shogaol

## Abstract

In the present study, the chemical investigation of the bioactive fractions of the rhizomes of *Zingiber officinale* has resulted in the identification of twenty-nine compounds including one new compound, *O*-methyldehydrogingerol (**1**). Some of the isolates were subjected into the evaluation of their antiplatelet aggregation and vasorelaxing bioactivities. Among the tested compounds, [6]-gingerol (**13**) and [6]-shogaol (**17**) exhibited potent anti-platelet aggregation bioactivity. In addition, [10]-gingerol (**15**) inhibited the Ca^2+^-dependent contractions in high K^+^ medium. According to the results in the present research, the bioactivity of ginger could be related to the anti-platelet aggregation and vasorelaxing mechanism.

## 1. Introduction

Ginger is the rhizome of *Zingiber officinale*, a perennial plant in the family Zingiberaceae. It is consumed as delicacy, medicine, or spice. It is often pickled in vinegar or sherry as a snack or just cooked as an ingredient in many dishes. There are a variety of uses suggested for ginger as folk medicines in Chinese history, such as stomachic, antiemetic, anti-diarrhea, expectorant, antiasthmatic, hemostatic and cardiotonic for the treatment of several gastrointestinal and respiratory diseases [1]. The most famous traditional medicinal practice of *Z. officinale* is to promote the blood circulation for removing blood stasis and the mechanism is related to anti-platelet aggregation activity [2]. In the course of our continuing research program aimed to discover novel bioactive constituents from natural sources, screening work for thrombolytic and vasoactive activity was carried out and the ether extract of the rhizome of *Z. officinale* was found to show anti-platelet aggregation activity and produce a vasorelaxing effect. This initiated our interest in studying the bioactive principles in the rhizome of *Z. officinale*. Numerous chemical investigations of the pungent and bioactive principles of *Z. officinale* have been carried out [3,4]. Among these compounds, [6]-gingerol is the major pungent principle of ginger and the chemopreventive potentials present a promising alternative to other toxic therapeutic agents [5]. In the present study, we report the characterization of twenty-nine compounds and the bioactivity of some of these purified compounds.

## 2. Results and Discussion

### 2.1. Characterization of the Isolated Compounds

**Figure 1:**
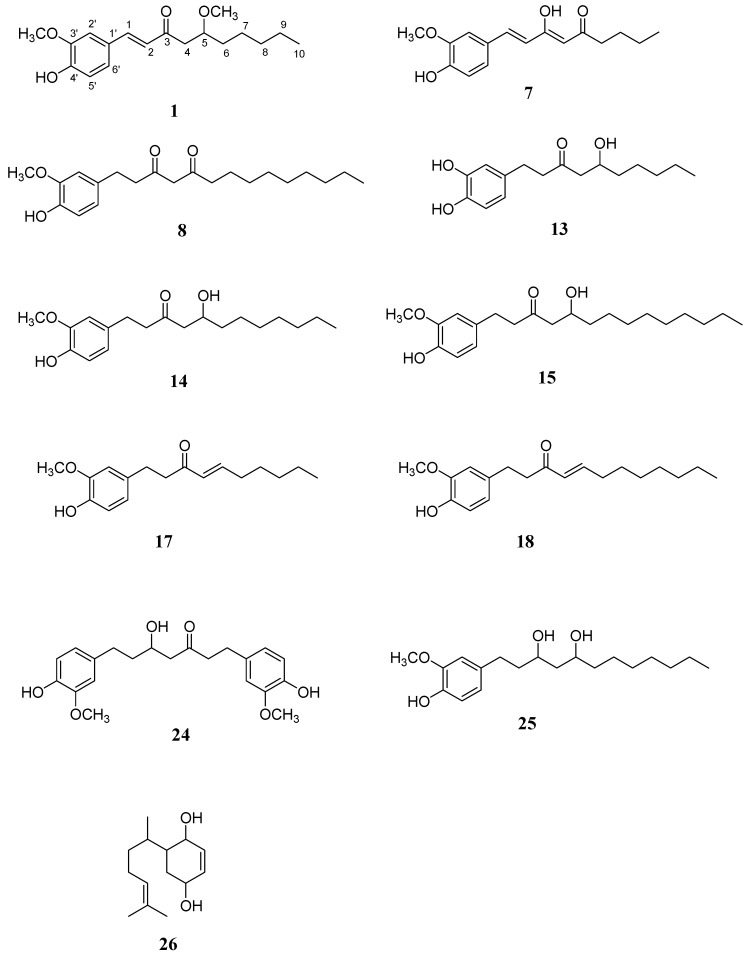
Structures of compound **1** and the purified constituents subjected into the bioactivity examinations (**7**, **8**, **13**–**15**, **17**, **18**, **24**–**26**).

Air-dried and chopped ginger was filtered and the filtrate was partitioned between ether and water. The residue was extracted with acetone and then also partitioned between ether and water. The ether fractions from the filtrate and residue were combined and concentrated to give a brown syrup. With the assistance of a combination of conventional chromatographic techniques, the ether extract of the *Z. officinale* afforded one new compound, *O*-methyldehydrogingerol (**1**), along with twenty eight known compounds, including 1-dehydro-[6]-dehydrogingerol (**2**) [6], *ar*-curcumen-15-al (**3**) [7], 3-(4-hydroxy-3-methoxyphenyl)propionic acid methyl ester (**4**) [8], curcumin (**5**) [9], 1*β*-hydroxy-bisabola-2,10-dien-4-one (**6**) [10], [6]-dehydrogingerdione (**7**) [11], [11]-isodehydrogingerdione (**8**) [12], [6]-, [8]-, and [10]-dehydroshogaol (**9**, **10**, and **11**), [6]-gingerdione (**12**), [6]-, [8]-gingerol (**13** and **14**) [13], [10]-gingerol (**15**), [4]-, [6]-, and [8]-shogaol (**16**, **17**, and **18**) [13], vanillin (**19**) [14], dihydrocurcumin (**20**) [9], methyl-[6]-gingerol (**21**) [15], *ar*-curcumene (**22**) [16], vanillic acid (**23**) [17], hexahydrocurcumin (**24**) [18], [6]-gingediol (**25**) [19], 2,5-dihydroxybisabola-3,10-diene (**26**) [20], glyceryl-1-hexadecanoate (**27**) [21], gingerenone-A (**28**) [22], and cryptomeridiol (**29**) [23]. The known compounds were identified by comparison of their physical and spectroscopic data with those reported in the literature. The chemical structure of new compound **1** was determined as 1-(4'-hydroxy-3'-methoxyphenyl)-5-methoxy-1-decen-3-one with the assistance of NMR and mass spectral analyses and it was given the trivial name *O*-methyldehydrogingerol. Among these isolated compounds, **7**, **8**, **13**–**15**, **17**, **18**, and **24**–**26** (Figure 1) were purified in more quantities and subjected to the bioactivity examinations. 

### 2.2. Anti-Platelet Aggregation Evaluation Bioassay

Platelets are a natural source of growth factors. They circulate in the blood of mammals and are involved in hemostasis, leading to the formation of blood clots. If the number of platelets is too high, blood clots would form which may obstruct blood vessels and result in some events such as stroke, myocardial infarction, and pulmonary embolism or the blockage of blood vessels to other parts of the body, such as the extremities of the arms or legs [24]. The traditional medicinal use of ginger is to promote the blood circulation for removing blood stasis and therefore the compounds isolated in more quantities (compounds **7**, **8**, **13**–**15**, **17**, **18** and **24**–**26**) were selected for the anti-platelet aggregation and vasorelaxing effect bioassay to evaluate their potential to be new lead drugs.

The anti-platelet aggregation results are summarized in Table 1. All the tested compounds displayed significant inhibitory effects on the aggregation of washed rabbit platelets stimulated by the agonists, including arachidonic acid (AA), collagen (Col), platelet activating factor (PAF), and thrombin (Thr). At 100 μg/mL concentration, all the tested compounds except **26** caused complete inhibition of aggregation induced by AA (100 μM). Compound **26** (100 μg/mL) also displayed 67.9% inhibition of the platelet aggregation. Even at lower concentration, compounds **7**, **8**, **13**, **17**, and **18**; **14** and **15**; **25**; and **24**, still exhibited excellent inhibitory activity (100%) against AA-induced platelet aggregation at 1.0, 2.0, 5.0, and 20.0 μg/mL, respectively. Compound **13** displayed the most significant inhibition of platelet aggregation induced by AA with inhibitory percentages of 83.7, 66.3, 16.7% at 0.5, 0.2, and 0.1 μg/mL, respectively. 

Similarly, compounds **7**, **8**, **13**, **14**, **15**, **17**, and **18** at 100 μg/mL showed the complete inhibition of aggregation induced by Col (10 μg/mL). However, the inhibitory activities of the tested compounds were not as effective as those induced by AA. At 5.0 μg/mL only compound **17** displayed excellent inhibition (100%) against Col-induced platelet aggregation. Compounds **13** and **17** exhibited the most significant inhibition of platelet aggregation induced by Col with inhibitory percentages of 15.6 and 28.1% at 1.0 μg/mL, respectively.

In contrast, the tested compounds did not show significant inhibition of aggregation induced by PAF (2 nM) and Thr (0.1 μg/mL). Only compound **17** at 100 μg/mL displayed complete inhibition of aggregation induced by PAF. At 20.0 μg/mL compound **17** only inhibited the platelet aggregation induced by PAF with the percentage of 15.7%. All the tested compounds at 100 μg/mL exhibited the inhibitory percentages lower than 29.5% against the platelet aggregation induced by Thr.

### 2.3. Vasorelaxing Effect Evaluation Bioassay

The results of inhibition of KCl- and norepinephrine (NE)-induced contraction of rat aorta were summarized in Table 2. All of the test compounds except **7** at high concentration showed significant inhibition of rat aorta tonic construction induced by Ca^2+^ (1.9 mM) in high K^+^ (80 mM) medium. It has been reported that high K^+^ induced contraction in vascular smooth muscle is mediated by an increase in Ca^2+^ influx through voltage-dependent Ca^2+^ channels [25]. Among the tested compounds, [10]-gingerol (**15**) depressed markedly the contractions induced, but not active against the phasic and tonic contractions caused by norepinephrine (3 μM). Since **15** inhibited the Ca^2+^-dependent contractions in high K^+^ medium, it may be a blocker of voltage-dependent Ca^2+^ channels.

**Table 1:** Effects of compounds **7**, **8**, **13**–**15**, **17**, **18**, **24**–**26** on the aggregation of washed rabbit platelets induced by arachidonic acid (AA), collagen (Col), PAF and thrombin (Thr).

Aggregation (%)												
Inducer	Control	Conc. (μg/mL)	7	8	13	14	15	17	18	24	25	26
AA	85.9 ± 0.6	0.1	-	-	83.3 ± 2.0				-	-	-	
(100 μM)		0.2	85.6 ± 0.2	-	33.7 ± 19.1 ^†^			80.6 ± 3.6	80.9 ± 1.7 ^†^	-	-	
		0.5	17.6 ± 12.5 ^‡^	82.9 ± 1.4	16.3 ± 10.1 ^‡^	82.1 ± 7.3	79.8 ± 3.8 ^†^	16.6 ± 14.4 ^‡^	1.4 ± 1.2 ^‡^	-	85.6 ± 1.7	
		1.0	0.0 ± 0.0 ^‡^	0.0 ± 0.0 ^‡^	0.0 ± 0.0^‡^	18.1 ± 3.8 ^‡^	2.7 ± 2.3 ^‡^	0.0 ± 0.0 ^‡^	0.0 ± 0.0 ^‡^	-	44.4 ± 21.1 *	
		2.0	-	-	-	0.0 ± 0.0 ^‡^	0.0 ± 0.0 ^‡^		-	86.1 ± 0.7	34.9 ± 18.3 ^‡^	
		5.0	-	-	-				-	41.5 ± 20.8 *	0.0 ± 0.0 ^‡^	
		10.0	-	-	-				-	27.9±16.4 ^‡^	-	
		20.0	-	-	-				-	0.0 ± 0.0 ^‡^	-	
		50.0	-	-	-				-	-	-	84.8 ± 0.5
		100.0	0.0 ± 0.0 ^‡^	0.0 ± 0.0 ^‡^	0.0 ± 0.0 ^‡^	0.0 ± 0.0 ^‡^	0.0 ± 0.0 ^‡^	0.0 ± 0.0 ^‡^	0.0 ± 0.0 ^‡^	0.0 ± 0.0 ^‡^	0.0 ± 0.0 ^‡^	32.1 ± 16.7 ^†^
Col	87.1 ± 1.0	0.5						83.3 ± 0.7 *	-	-		
(10 μg/mL)		1.0	-	-	84.4 ± 2.9			71.9 ± 4.7 ^‡^	-	-		
		2.0	78.5 ± 2.7	-	69.6 ± 9.9 ^†^		84.2 ± 3.7	30.6 ± 10.8 ^‡^	82.5 ± 4.0			
		5.0	37.4 ± 15.6 ^‡^	86.3 ± 1.3	40.6 ± 17.9 ^‡^	76.8 ± 4.8 ^†^	77.7 ± 1.9 ^‡^	0.0 ± 0.0 ^‡^	25.4 ± 14.0 ^‡^			
		10.0	4.6 ± 4.0 ^‡^	54.8 ± 18.0 *	12.6 ± 10.3 ^‡^	29.0 ± 16.9 ^‡^	20.6 ± 10.6 ^‡^		0.0 ± 0.0 ^‡^		80.1 ± 5.1	
		20.0	0.0 ± 0.0 ^‡^	4.6 ± 4.0 ^‡^	0.0 ± 0.0 ^‡^	0.0 ± 0.0 ^‡^	0.0 ± 0.0 ^‡^			77.6 ± 4.0 ^†^	54.7 ± 15.6 *	
		50.0	-	0.0 ± 0.0 ^‡^	-					33.0 ± 17.2 ^‡^	47.2 ± 13.7 ^‡^	
		100.0	0.0 ± 0.0 ^‡^	0.0 ± 0.0 ^‡^	0.0 ± 0.0 ^‡^	0.0 ± 0.0 ^‡^	0.0 ± 0.0 ^‡^	0.0 ± 0.0 ^‡^	0.0 ± 0.0 ^‡^	3.8 ± 3.3 ^‡^	15.5 ± 8.9 ^‡^	89.5 ± 1.8
PAF	88.6 ± 1.8	20.0						84.3 ± 3.6				
(2 nM)		50.0	83.9 ± 1.0	83.0 ± 1.0 *		83.2 ± 1.4	83.4 ± 1.6	59.9 ± 8.5 ^†^	84.9 ± 4.0			
		100.0	55.8 ± 4.7 ^‡^	55.3 ± 11.6 ^†^		24.4 ± 8.6 ^‡^	10.5 ± 8.6 ^‡^	0.0 ± 0.0 ^‡^	55.8 ± 9.2 ^†^	84.5 ± 2.4	84.9 ± 1.5	85.6 ± 1.3
Thr (0.1 μg/mL)	92.3 ± 1.0	100.0	87.4 ± 1.1	84.5 ± 0.6 ^‡^		85.0 ± 0.5 ^‡^	84.3 ± 2.5 *	70.5 ± 2.7 ^‡^	80.2 ± 0.9 ^‡^	85.1 ± 4.7	88.5 ± 1.1 *	88.2 ± 3.6

Platelets were preincubated with compounds or DMSO (0.5%, control) at 37 °C for 3 min; the inducer was then added. Values were means ± s.e.m. (n = 3–7). * *p* < 0.05, ^†^ *p* < 0.01, ^‡^*p* < 0.001 as compared with the control.

**Table 2:** Inhibition of KCl, norepinephrine-induced contraction of rat aorta by compounds **7**, **8**, **13**–**15**, **17**, **18**, **24**–**26**.

Compound	Conc. (μM)	NE (3 μM)contraction (%)		K^+^ (80 mM) + Ca^2+^ (1.9 mM)contraction (%)
		Phasic	Tonic	Tonic
Control		100.0 ± 5.1	100.0 ± 4.1	100.0 ± 4.9
**7**	362	86.6 ± 1.6	84.7 ± 1.5	75.4 ± 3.2
**8**	287.4	100.0 ± 0.0	85.3 ± 2.8	46.9 ± 4.2
	86.2	–	–	72.3 ± 3.7
**13**	357.1	49.2 ± 2.3	40.3 ± 3.8	13.4 ± 1.1
	107.1	88.7 ± 1.9	93.3 ± 0.0	26.4 ± 2.4
	35.7	–	–	96.7 ± 1.9
**14**	310.6	63.3 ± 3.1	48.1 ± 3.9	12.5 ± 0.0
	93.2	89.9 ± 9.2	73.2 ± 2.8	20.9 ± 2.1
	31.1	–	–	83.2 ± 4.9
**15**	285.7	104.2 ± 1.9	65.1 ± 7.2	23.8 ± 3.1
	85.7	–	–	48.1 ± 0.9
	28.6	–	–	68.6 ± 4.9
**17**	362.3	60.0 ± 14.1	36.7 ± 2.4	11.0 ± 0.4
	108.7	90.3 ± 0.9	89.9 ± 1.7	29.7 ± 3.8
	36.2	–	–	83.0 ± 1.1
**18**	328.9	109.7 ± 3.7	78.6 ± 2.8	24.3 ± 4.3
	98.7	–	–	58.0 ± 1.9
	32.9	–	–	93.2 ± 1.9
**24**	267.4	76.7 ± 10.0	72.2 ± 3.2	50.9 ± 7.8
	80.2	–	–	95.0 ± 4.9
**25**	308.6	78.3 ± 2.3	52.7 ± 2.9	12.0 ± 1.0
	92.6	83.9 ± 1.8	83.5 ± 4.1	71.7 ± 1.8
**26**	446.4	72.5 ± 1.8	53.0 ± 1.3	45.1 ± 5.1
	133.9	80.0 ± 0.0	75.3 ± 3.8	88.6 ± 7.8

Rat aorta rings were preincubated with compounds or DMSO (1%, control) at 37 °C for 15 min, the inducer was then added. Values were means ± s.e.m. “–”: no test.

### 2.4. Structure-Bioactivity Relationships

From the above bioassay data, [6]-gingerol (**13**) and [6]-shogaol (**17**) exhibited more potent anti-platelet aggregation bioactivity. We could conclude that the compounds which possessed a carbonyl groupa at the 6-position of the alkyl chain in phenylalkanoids and phenylalkenoids would be good drug lead candidates. According to the purified constituents and bioactivity in the present research, the function of ginger to promote the blood circulation for removing blood stasis could be related to anti-platelet aggregation and vasorelaxing mechanism.

## 3. Experimental

### 3.1. General Procedures

All the chemicals were purchased from Merck KGaA (Darmstadt, Germany) unless specifically indicated. Melting points of purified compounds were determined by a Yanagimoto micromelting point measuring apparatus (Tokyo, Japan) without corrections. The UV spectra were obtained on a Hitachi UV-3210 spectrophotometer (Tokyo, Japan). The IR spectra were obtained as KBr discs on a Jasco Report-100 FT-IR spectrometer (Tokyo, Japan). Optical rotations were measured with the Jasco DIP-370 Digital polarimeter (Tokyo, Japan). The mass and high-resolution mass spectra were obtained on a VG-70-250S mass spectrometer equipped with a direct inlet system. ^1^H-, ^13^C-, and 2D-NMR spectra were recorded on the Bruker AC-200, AMX-400 (Bruker Biospin Inc., Ettlingen, Germany) and Varian Unity (Palo Alto, CA, USA) plus 400 MHz spectrometers with tetramethylsilane as the internal standard. Standard pulse sequences and parameters were used for the NMR experiments and all chemical shifts were reported in parts per million (ppm, δ). Column chromatography was performed on silica gels (Kieselgel 60, 70–230 mesh and 230–400 mesh, Merck KGaA, Darmstadt, Germany). Thin layer chromatography (TLC) was conducted on precoated Kieselgel 60 F 254 plates (Merck) and the compounds were visualized by UV light or spraying with 10% (v/v) H_2_SO_4_ followed by heating at 110 °C for 10 min. 

### 3.2. Isolation

The chopped rhizomes of *Z. officinale* (64.4 kg) were filtered to afford the ginger juice and the residue. The filtrate was partitioned between ether (Et_2_O) and water (H_2_O), whereas the residue was extracted with acetone five times. The acetone extracts were combined, concentrated, and then also partitioned between Et_2_O and H_2_O. The ether solutions from the filtrate and residue were combined and concentrated again to give a brown syrup (623 g) which was subjected to silica gel column chromatography using a gradient of benzene (C_6_H_6_) and acetone (Me_2_CO) as eluent to yield twelve fractions. Frs 1–4 were mixed together and rechromatographed over silica gel developing with C_6_H_6_-Me_2_CO (9:1) to give **1** (5 mg), **2** (2 mg), **7** (295 mg), **8** (4 mg), and **12** (7 mg), successively. The combination of Frs 5–8 was repeated chromatographed over silica gel and thin layer chromatography (TLC) to yield **3** (2 mg), **4** (2 mg), **6** (3 mg), **9** (7 mg), **10** (3 mg), **11** (2 mg), **13** (18.6 mg), **14** (675 mg), **15** (96 mg), **17** (18 mg), **18** (7 mg), **19** (2 mg), **20** (23 mg), and **21** (22 mg) from the subfractions, respectively. Frs 9–11 were also combined and rechromatographed over silica gel eluting with CHCl_3_-Me_2_CO (9:1) to afford **16** (4 mg), **22** (2 mg), **23** (5 mg), **24** (530 mg), **25** (2 mg), **26** (60 mg), **27** (2 mg) and **28** (3 mg), successively. The last fraction was rechromatographed on RP-18 column eluting with H_2_O and step gradient of methanol to yield **5** (2 mg) and **29** (3 mg).

### 3.3. Antiplatelet Aggregatory and Vasorelaxing Activity Bioassays

Assays of the antiplatelet aggregatory and vasorelaxing activities of isolates were done according to the procedures of Teng and coworkers [26,27]. Washed platelets were prepared from blood withdrawn with a siliconized syringe from the marginal vein of New Zealand rabbits. The platelet suspension was obtained from EDTA-anticoagulated platelet-rich plasma according to the washing procedure described previously. Platelet number was counted with a cell counter (Hema-laser 2, Sebia, France) and adjusted to 3.0 × 10^8^ platelets/mL. The platelet pellets were suspended in Tyrode’s solution containing Ca^2+^ (1 mM) and bovine serum albumin (0.35%). All glassware was siliconized. Platelet aggregation was measured by the turbidimetric method [27]. The aggregations were measured with a Lumi-aggregometer (Model 1020, Payton, Stouffville, Canada) connected to two dual-channel recorders.

### 3.4. Spectral Data

*O-Methyldehydrogingerol* (**1**): colorless syrup; [α]_D_ −30.2° (c 0.02, CHCl3); UV λmax 228, 290 (sh), 341 nm; IR νmax 3450, 1651, 1589, 1515 cm^−1^; ^1^H-NMR (CDCl3, 400 MHz) δ 0.88 (3H, t, *J* = 6.5 Hz, H-10), 1.2–2.4 (8H, m, H-6 to H-9), 2.65 (1H, dd, *J* = 15.6, 5.2 Hz, H-4a), 2.95 (1H, dd, *J* = 15.6, 7.6 Hz, H-4b), 3.35 (3H, s, OCH_3_-5), 3.77 (1H, m, H-5), 3.94 (3H, s, OCH_3_-3'), 5.90 (1H, br s, OH-4'), 6.63 (1H, d, *J* = 16.0 Hz, H-2), 6.93 (1H, d, *J* = 8.0 Hz, H-5'), 7.06 (1H, d, *J* = 2.0 Hz, H-2'), 7.11 (1H, dd, *J* = 8.0, 2.0 Hz, H-6'), 7.50 (1H, d, *J* = 16.0 Hz, H-1); ^13^C NMR (CDCl3, 100 MHz) δ 14.1 (C-10), 22.6 (C-9), 29.1 (C-7), 29.5(C-8), 32.5 (C-6), 41.7 (C-4), 55.9 (OCH_3_-3'), 56.3 (OCH_3_-5), 76.8 (C-5), 114.4 (C-1'), 119.6 (C-2'), 127.8 (C-5'), 130.2 (C-2), 134.5 (C-6'), 144.8 (C-1), 145.4 (C-4'), 147.7 (C-3'), 199.6 (C=O); EIMS *m/z* (*rel. int.*) 306 (M^+^, 21), 235 (9), 177 (100), 150 (53), 145 (18), 137 (17), 117 (9), 55 (12); HREIMS *m/z* 306.1838 [M]^+^ (calcd for C_18_H_26_O_4_, 306.1831).

## 4. Conclusion

In summary, twenty-nine compounds were isolated from the rhizomes the *Z. officinale*. The pharmacological activities including anti-platelet aggregation and vasorelaxing effects have been documented. However, dose and the form in which they should be used require further standardization. All these are due to the synergistic effects of zingiberene and related types of components bring about the pharmacological impact. In conclusion, ginger has a wide range of medicinal uses and can be used either as a single drug or compound drugs to treat different ailments. 
